# Erythropoietin-derived peptide treatment reduced neurological deficit and neuropathological changes in a mouse model of tauopathy

**DOI:** 10.1186/s13195-020-00766-4

**Published:** 2021-01-27

**Authors:** Yun-Beom Choi, Ambrose A. Dunn-Meynell, Michelle Marchese, Benjamin M. Blumberg, Deeya Gaindh, Peter C. Dowling, Wei Lu

**Affiliations:** 1 Neurology Service, VA New Jersey Health Care System and Department of Neurology, Rutgers New Jersey Medical School, 385 Tremont Ave., East Orange, NJ 07018 USA; 2 Neurology Service, VA New Jersey Health Care System and Department of Pharmacology, Physiology, and Neuroscience, Rutgers New Jersey Medical School, 385 Tremont Ave., East Orange, NJ 07018 USA; 3 Neurology Service, VA New Jersey Health Care System, 385 Tremont Ave., East Orange, NJ 07018 USA

**Keywords:** Tauopathy, Alzheimer’s disease, Mouse model, Erythropoietin, Neuroinflammation, Microglia

## Abstract

**Background:**

Prominent activation of microglial immune/inflammatory processes is a characteristic feature of brains of patients with tauopathies including Alzheimer’s disease (AD), suggesting that neuroinflammation may be a critical factor in their pathogenesis. Strategies aimed at developing new therapeutics for tauopathies based on anti-inflammation or immunomodulation are likely to be promising avenues of research. We previously developed JM4—a 19’mer cyclic peptide derived from the first loop of human erythropoietin. This peptide possesses beneficial immune modulatory and tissue protective effects while lacking the undesirable side effects of full-length erythropoietin. In this preclinical study, we investigated the effect of chronic JM4 treatment on the PS19 mouse that carries the P301S mutant human tau gene, linked to a form of frontotemporal dementia. This transgenic mouse has been widely used as a model of tauopathies including AD and related dementias.

**Methods:**

Daily subcutaneous treatment of female PS19 mice with JM4 was initiated before disease onset and continued on for the animals’ lifespan. The progression of neurological deficit and the lifespan of these mice were assessed. To evaluate the effect of JM4 treatment on cognition of these animals, the PS19 mice underwent Barnes maze test and elevated plus maze test. In addition, neuronal loss, phosphorylated tau aggregation, and microglial activation were assessed using immunohistochemistry of PS19 mouse brain sections.

**Results:**

JM4 treatment of PS19 mice initiated before disease onset reduced neurological deficit, prolonged lifespan, and rescued memory impairment. The beneficial effects of JM4 were accompanied by reductions in neuronal loss, phosphorylated tau aggregation, and microglial activation in the PS19 mouse brain.

**Limitations:**

Use of a single dose of JM4 and female mice only.

**Conclusion:**

JM4 is a potential novel therapeutic agent for the treatment of tauopathies including AD and related dementias.

## Background

Prominent activation of a microglial immune/inflammatory process is a characteristic feature in the brains of patients with Alzheimer’s disease (AD) and related dementias, suggesting that neuroinflammation may be a critical factor in their pathogenesis [1–3]. Supporting this idea, it has been shown that inflammatory cells such as microglia and inflammatory mediators including complement components and proinflammatory cytokines were elevated in the brains of patients with AD and related dementias [4]. Microglia are the resident immune cells in the central nervous system (CNS) and appear to be a key player in the process as neuroinflammation in these neurodegenerative diseases primarily involves the innate immune system [5, 6]. One of the main molecular pathways through which microglia play a role in AD pathogenesis is the complement cascade; this conclusion is based on recent studies showing that complement components, C1q, C3, and C3R are enriched at the synapse and play a direct role in synapse removal by microglia in animal models of AD and related dementias [7–10]. Strategies aimed at developing new therapeutics for AD and related dementias based on anti-inflammation or immunomodulation mediated by microglia are likely to be promising avenues of research [11–13].

Neurofibrillary tangles (NFT) composed of insoluble fibrillary aggregates of hyperphosphorylated tau, a microtubule-associated protein, are neuropathological hallmarks of AD [14]. In addition to AD, tau pathology represented by NFT is found in other neurodegenerative diseases including frontotemporal dementia (FTD), progressive supranuclear palsy, and corticobasal degeneration, collectively known as tauopathies [15]. Currently, there is strong evidence for involvement of microglia in the pathology of tauopathies including AD. For example, there is a positive correlation between NFT burden and the number of activated microglia in a postmortem study of AD brains [16]. In animal models, exacerbation of inflammation increases tau pathology, while diminishing inflammation has the opposite action [17, 18]. Furthermore, activated microglia triggers hyperphosphorylation and aggregation of tau, which may be blocked by antagonizing the proinflammatory cytokine IL1β produced by microglia [19].

In our laboratory's search for a potential anti-inflammatory/immunomodulatory agent for tauopathy therapeutics, we turned our focus to a peptide derivative of erythropoietin (EPO). EPO is a cytokine that is essential for red blood cell production [20]. It has been shown to be neuroprotective in the CNS through multiple cellular mechanisms including anti-inflammatory/immunomodulation, blockade of apoptosis, and anti-oxidant signaling [21–24]. However, EPO’s potential as a therapeutic agent in humans has been compromised because its induction of hematopoiesis may lead to increased risk of morbidity and mortality from stroke and cardiovascular events due to thromboembolism [25]. The crystal structure of the EPO–EPO receptor complex shows that one EPO molecule forms a compact globular structure containing a four α–helical bundle (αA-αD) topology [26]. One EPO molecule interacts with two receptor molecules forming a homodimer via two binding sites located on opposite faces of the EPO (sites 1 and 2); site 1 consists of a portion of αD and a loop connecting αA and αB whereas site 2 consists of portions of αA and αC [26]. It has been suggested that the regions within the EPO molecule that do not involve EPO receptor biding may mediate the non-hematopoietic tissue-protective effects, perhaps binding to a receptor different from a EPO receptor homodimer, but this issue is still controversial [27, 28]. Regardless, derivatives of EPO that possess the beneficial immunomodulatory and tissue protective effects but lack the undesirable hematopoietic activities have been developed and assessed as therapeutic agents in animal models of various neurological disorders including exogenously applied beta-amyloid induced pathology [27, 29–32]. Yet, the effects of EPO or its derivative in animal models of tauopathy have not been investigated.

Following this line of reasoning, our laboratory developed JM4, a 19’mer peptide derived from the first loop of human EPO corresponding to amino acid sequence 28 to 46 and cyclized for stability [33]. JM4 sequence is located at a loop connecting αA and αB comprising a part of site 1 of EPO binding to EPO receptor. JM4 was highly effective in reducing neurologic deficit in experimental autoimmune encephalomyelitis (EAE) and in a murine acute traumatic brain injury model without inducing hematocrit alterations [33, 34]. In EAE mice, this low molecular weight peptide appears to work through downregulation of the innate immune system and through downstream elements of the adaptive immune system [33]. In a controlled cortical impact model of acute traumatic brain injury in mice, JM4 treatment within 15 min to 9 h after the brain trauma blocked neuronal death [34]. Importantly, JM4 readily crosses the blood-brain barrier into the CNS [34].

In this preclinical in vivo study, we investigated the therapeutic potential of JM4 in a mouse model of tauopathy. Specifically, we used the PS19 transgenic mouse that expresses the P301S mutant human tau associated with FTD and Parkinsonism linked to chromosome 17 [18]. In these mice, tau aggregates develop at 6 months of age and progressively accumulate in association with neuronal loss by 9–12 months of age [18]. Moreover, prominent microglial activation has been detected well before tau aggregates emerge in PS19 mice [18]. These mice exhibit behavioral abnormalities recapitulating deficits seen in human tauopathies [35]. We show that daily subcutaneous treatment with JM4 initiated before disease onset reduced neurologic deficits, prolonged lifespan, rescued memory impairment, and decreased neuronal loss, NFT accumulation, and microglial activation in PS19 mice. Along with our recently published brief supplementary study of assessing the effect of JM4 treatment on PS19 mice noninvasively using bioluminescence imaging of glia fibrillary acid protein [36], this is the first study showing beneficial effects of EPO or its derivatives in a mouse model of tauopathy.

## Methods

### Animals

We used the transgenic mouse strain, PS19, B6;C3GTg(PrnpGMAPT*P301S)PS19Vle/J (Jackson Laboratory, Bar Harbor, ME), which is hemizygous for the P301S mutation in human tau gene, *MAPT*, driven by the prion protein (PrnP) promoter. Only female mice were used to avoid potential sex differences in response to treatment. This mouse strain has been well characterized [18, 35]. The mice were housed under a 12-h light/dark cycle and given ad libitum access to food and water. The studies were conducted in accordance with the Animal Component of Research Protocol approved by Institutional Animal Care and Use Committee (IACUC) at the VA New Jersey Health Care System, East Orange, NJ, and with the United States Public Health Service’s Policy on Humane Care and Use of Laboratory Animals.

### JM4 treatment

As previously described, JM4 (sequence: GCAEHCSLNENITVPDTKV) was synthesized using solid phase techniques, and then purified by high performance liquid chromatography (HPLC) to more than 90% purity (United Biochemical Research, Seattle, WA) [33, 34]. Sample purity was established by MALDI-TOF mass spectrometry. JM4 was dissolved in phosphate-buffered saline (PBS) (1 mg/ml) and kept in small aliquots at − 20 °C until use. JM4 was injected at 10 μg/day/mouse subcutaneously, 5 days a week. The injection position was rotated to prevent skin reactions at the injection site.

### Neurological assessment

Mice were scored for neurological deficit by examining the limb retraction and clasping found in this transgenic mouse model to determine the time of disease onset. The neurological assessment is a modified version of the limb clasping test described by Miller et al. [37]. Briefly, the mouse was suspended by its tail for 30 s and a clinical score was assigned on the following scale: 0 = no functional deficit, 1 = limb clasping lasting over 10 s, 2 = retraction of one or more limbs for the full 30 s, 3 = failure to lift the body due to weakness, 4 = total hind limb paralysis; and 5 = paralysis of all 4 limbs. Two examiners blinded to the experimental treatment carried out the neurological examination and an average of the two observations was used. Disease onset was defined by the time at which a mouse reaches a clinical score of 2. Mice were then monitored until close to end of life. Criteria including difficulty drinking/eating, limited mobility, and weight loss greater than 30% were used to determine when mice had reached their humane end point. The end of life data allowed us to develop Kaplan-Meier survival curves.

### Barnes maze test

Mice were placed in the center of a white circular surface, 1.2 m diameter, with 40 holes (5 cm in diameter) around the perimeter. The circular open field was elevated 1 m from the floor. Then, mice were expected to learn the location of a target hole, under which a dark escape box was located. Bright lights (four 100 W bulbs placed 4 ft above the maze) were used as negative reinforcements to complete the task. Four different simple colored-paper shapes were mounted around the room as visual cues. During the memory acquisition phase, the mice were allowed to explore the maze for 3 min per trial (3 trials per day) for 4 consecutive days. Twenty-four hours after the last training, spatial memory was tested with a probe test in which the mice had 2 min to find the target hole. Time to find the target hole and distance-traveled were measured to assess spatial memory. Behavior was recorded and analyzed using the Noldus Ethovision XT tracking software (Noldus Information Technology, Leesburg, VA).

### Elevated plus maze test

We used a plus-shaped (+) apparatus, which had two open and two side-shielded arms (each 35 cm × 5.5 cm) as well as an open central platform (5.5 cm × 5.5 cm) elevated 60 cm above the ground. Mice were acclimatized to the room for 1 h prior to testing, and then placed on the central platform facing an open arm. Mouse behavior was recorded during a 5-min test period. The percentage of time spent on the open arms and distance-traveled were measured to assess anxiety-like behavior. Behavior was recorded and analyzed using the Noldus Ethovision XT tracking software (Noldus Information Technology, Leesburg, VA).

### RNA extraction and quantitative reverse-transcription polymerase chain reaction (RT-PCR)

Total RNA was extracted from one hemisphere of mouse brain tissue using RNeasy Mini kit (QIAGEN, Germantown, MD). Reverse transcription was carried out using Superscript IV First Strand synthesis system (Invitrogen, Waltham, MA). The quantitative RT-PCR analyses were performed on an Applied Biosystems 7500 Real-Time PCR System (Applied Biosystems, Waltham, MA), using FastStart SYBR Green Master Mix (Roche, Indianapolis, IN). Primer sequences are human *Mapt* (tau): forward GTCGAAGATTGGGTCCCT, reverse GACACCACTGGCGACTTGTA, mouse *Gapdh* (glyceraldehyde 3-phosphate dehydrogenase): forward CATCACTGCCACCCAGAAGACTG and reverse ATGCCAGTGAGCTTCCCGTTCAG, mouse *Iba-1* (ionized calcium binding adapter molecule 1): forward CAGACTGCCAGCCTAAGACA and reverse AGGAATTGCTTGTTGATCCC, mouse *Cd68*: ACTGGTGTAGCCTAGCTGGT, CCTTGGGCTATAAGCGGTCC, mouse *Il1β* (interleukin 1 beta): forward GCAACTGTTCCTGAACTCAACT and reverse ATCTTTTGGGGTCCGTCAACT, mouse *Il6* (interleukin 6): forward TAGTCCTTCCTACCCCAATTTCC and reverse TTGGTCCTTAGCCACTCCTTC, and mouse *Tnfα* (tumor necrosis factor alpha): forward CCCTCACACTCAGATCATCTTCT and reverse GCTACGACGTGGGCTACAG. *Gapdh* was used as a reference gene to normalize for the amount of mRNA. Samples were run in triplicate. The analysis of relative gene expression was performed using ΔΔCt (threshold cycle) method.

### Brain extraction and Western blot

Mice were anesthetized deeply with ketamine (80 mg/kg) and xylazine (7.5 mg/kg) and then transcardially perfused with PBS. Brains were frozen on dry ice immediately and stored at − 80 °C before homogenization. Brain samples were weighed and homogenized in RAB (reassembly) buffer (100 mM MES, 1 mM EGTA, 0.5 mM MgSO_4_, 0.75 M NaCl, 20 mM NaF; G-Biosciences) with protease and phosphatase inhibitor mix (Roche) at 10 μL buffer per milligram brain by T10 Dispersion unit using 5G dispenser at the max setting for 10 s for 3 times. Then, brain homogenate suspensions were centrifuged at 50,000*g* for 20 min at 4 °C. The supernatant was collected and stored at − 80 °C as RAB fraction (aqueous fraction). The pellet was resuspended with RIPA (radioimmunoprecipitation assay) buffer (25 mM Tris, 150 mM Sodium Chloride, 1% NP-40, 1% Sodium Deoxycholate, 0.1% SDS, pH 7.6; G-Biosciences) with protease and phosphatase inhibitor mix (Roche) at 10 μL buffer per milligram brain by sonicating 30 s for 2 time setting out-power at 80%. Then, the suspension was centrifuged at 50,000*g* for 20 min at 4 °C. The supernatant was collected and stored at − 80 °C as RIPA fraction (detergent soluble fraction). The pellets were further lysed in 0.5 mL urea buffer (8 M urea and 5% (w/v) SDS, pH 7.2) by sonicating 30 s for 2 times setting out-power at 80%. Then, the suspension was centrifuged at 50,000*g* for 20 min at 4 °C. The supernatant was collected and stored at − 80 °C as urea fraction (insoluble fraction). Each protein sample (25 μg) was separated by 12% SDS-PAGE and transferred onto 0.45 μm PVDF membranes (GE healthcare) and then incubated with 10% no fat-milk overnight with gentle agitation at 4 °C. For phosphorylated tau, the blots were incubated with primary antibody (AT8, Thermo Scientific, MN1020) at 1:300 dilution for 1 h at room temperature followed by incubation with a secondary antibody (horse radish peroxidase (HRP)-conjugated anti-mouse, GE Healthcare) at 1:3000 dilution for 1 h at room temperatures. For glyceraldehyde 3-phosphate dehydrogenase (GAPDH) loading control, the blots were incubated with a rabbit anti-mouse GAPDH-HRP antibody (Abcam, AB201822) at 1:20,000 dilution for 1 h at room temperature. Blots were incubated with ECL detection reagents and images obtained under a chemiluminescent imaging system.

### Immunohistochemistry

Mice were deeply anesthetized deeply with ketamine (80 mg/kg) and xylazine (7.5 mg/kg) and then transcardially perfused with 4% paraformaldehyde in PBS. The brains were removed and immersed in the same fixative at 4 °C for 12 h and then cryoprotected in 20% sucrose in PBS overnight. Brains were frozen and coronal sections were serially cut at 8 μm-thick and collected on slides, then post-fixed in acetone at − 20 °C for 10 min. Sections were first treated with Target Unmasking Fluid (Pan Path) at 90 °C for 10 min and blocked in 2% normal horse serum for 10 min. Sections were then incubated overnight at 4 °C with the following primary antibodies: monoclonal mouse anti-human PHF-tau pSER202/Thr205 antibody (AT8, Thermo Scientific, MN1020) at 1:20 dilution, monoclonal rat anti-mouse 1-A/1-E clone MS/144 (BD Pharmingen, #556999) at 1:200 dilution, polyclonal rabbit anti-NeuN (Millipore, ABN78) at 1:500 dilution, monoclonal mouse anti-human tau (TAU5, BioLegend, #806401) at 1:50 dilution, polyclonal rabbit anti-IBA-1 (Wako, #01919741) at 1:500 dilution, and monoclonal rat anti-mouse C3 complement (Abcam, AB11862) at 1:50 dilution. The following day, sections were incubated with cy3- or cy2- conjugated goat anti-mouse, anti-rat, or anti-rabbit IgG (Jackson ImmunoResearch) at 1:120 dilution for 1 h. Sections were then washed in dH_2_O three times for 10 min each, followed by fluorescence microscopy imaging. Brains of mice that reached the end of life before 10 months of age were not included in the analysis of immunohistochemistry data.

### Fluorescence microscopy imaging

Immunohistochemistry images were obtained using an Olympus BX41 fluorescent microscope (Center Valley, PA) fitted with ProgRes MF cool digital camera using × 2 or × 10 objectives. We imaged 3–5 sections per animal. Images were quantified by measuring the percent area in the region of interest, where label exceeded a constant threshold value and by counting the number of NeuN+ cells per field of view, using Image Pro Premier 9.3 image analysis software (BD Biosciences, Franklin Lakes, NJ).

### Statistics

For analysis of disease onset and survival data, Kaplan-Meier plots followed by the Mantel-Cox log rank test were used. For behavioral testing, immunohistochemistry, and quantitative RT-PCR data, we used unpaired *t* test for two-group comparison and one-way ANOVA followed by Tukey’s tests for every pair of group comparisons or Bonferroni test for selected pairs of group comparisons. Grubbs’ Test was performed on the immunohistochemistry and quantitative RT-PCR data to identify and to remove outliers before statistical analysis. All statistical analyses were performed with Prism 5.0 software (GraphPad, La Jolla, CA). Data were expressed as mean ± standard error. Rectangular boxes around the data points were added for visual enhancement of the data ranges in figures except Fig. 5g, which is a bar graph.

## Results

### Chronic JM4 treatment reduced neurological deficit and prolonged lifespan in PS19 mice

**Fig. 1:**
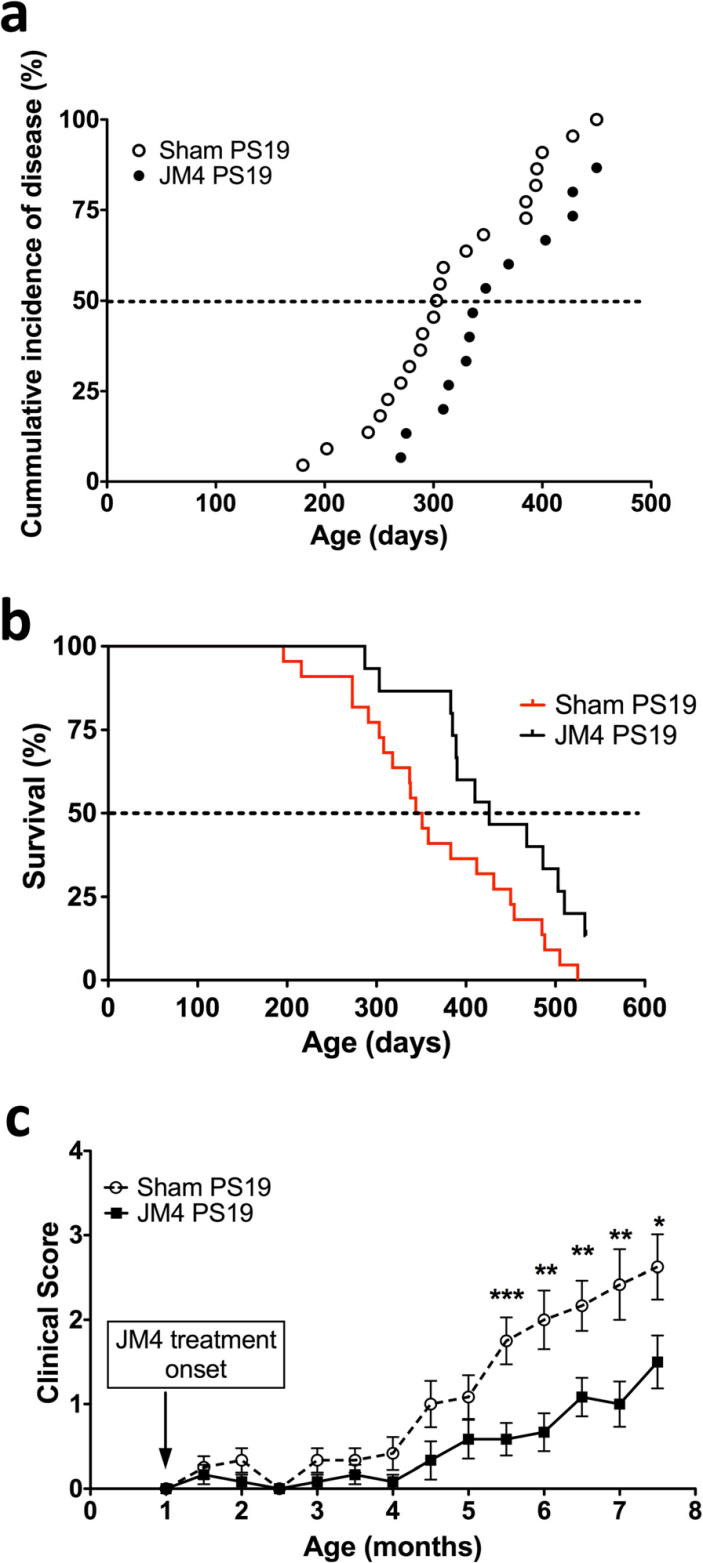
Chronic JM4 treatment reduced neurological deficit and prolonged lifespan in PS19 mice. **a** In a cohort of mice in which JM4 treatment was initiated at 2 months of age, the PBS sham-treated PS19 mice (open circles) showed an earlier onset of disease than the JM4-treated PS19 mice (filled circles). Disease onset was defined as the age at which mice displayed clasping of at least one hind limb for 30 s when suspended by their tail (clinical score 2) (*p* = 0.03, log rank (Mantel-Cox) test). **b** Kaplan-Meier curves show that JM4 treatment (black line) prolongs lifespan of PS19 mice compared to PBS sham-treatment (red line) (*p* = 0.02, log rank (Mantel-Cox) test). **c** In another cohort of mice in which JM4 treatment was initiated at one month of age, JM4-treated PS19 mice (filled squares) display less severe neurological deficit compared to PBS sham-treated PS19 mice (open circles) from 5.5 months and on (*p < 0.05, **p < 0.01, two-tailed unpaired *t* test for each time point)

A cohort of PS19 mice was treated with JM4 (10 μg for 5 days/week, subcutaneously) initiated at 2 months of age, before the onset of neurological deficit, to assess the effect of JM4 treatment on the onset of disease and the lifespan of mice. Neurological deficit was assessed daily on a scale of 0 (no deficit) to 5 (severe deficit). The onset of neurological disease was defined as the age at which a mouse reached a clinical score of 2. A clinical score of 2 corresponds to a mouse displaying clasping of at least one hind limb for 30 s when suspended by its tail. JM4 treatment (*n* = 15) significantly delayed the onset of neurological disease compared to the PBS sham-treated group (*n* = 22) (median onset of disease = 348 days for JM4-treated group vs. 305 days for PBS sham-treated group) (Fig. 1a). This cohort of mice was then monitored until end of life. The end of life data allowed us to develop Kaplan-Meier survival curves, which showed that JM4 treatment prolonged survival of PS19 mice compared to the PBS sham-treated group (median lifespan 447 days, for JM4-treated group vs. 348 days for PBS sham-treated group) (Fig. 1b). Note that this cohort of PS19 mice had a longer lifespan compared to the median survival of about nine months of PS19 mice reported initially by Yoshiyama et al. [18]. However, it has been reported that the onset and severity of tau pathology in PS19 mice are rather variable [38].

Another cohort of PS19 mice was treated with JM4 (10 μg for 5 days/week, subcutaneously) initiated at 1 month of age before the onset of neurological deficits. Every half month, neurological deficit was scored on a scale of 0 (no deficit) to 5 (severe deficit). We found JM4 treatment significantly reduced neurological deficit in PS19 mice compared to a group sham-treated with PBS (*n* = 12 for each group) (Fig. 1c). Because neurological deficit in PS19 mice was minimum up to 5 months of age, the difference in clinical scores between JM4-treated group and sham-treated group only became significant from 5.5 months of age onward. This cohort of mice was subjected to behavioral testing and subsequent neuropathological assessment.

### Chronic JM4 treatment rescued behavioral abnormalities in PS19 mice

PS19 mice display cognitive impairment, including selective deficits in spatial learning and memory at 6 months of age [35]. A subset of mice that were treated with JM4 from 1 month old underwent several behavioral tests to assess the effect of JM4 treatment on cognition of these animals.

**Fig. 2:**
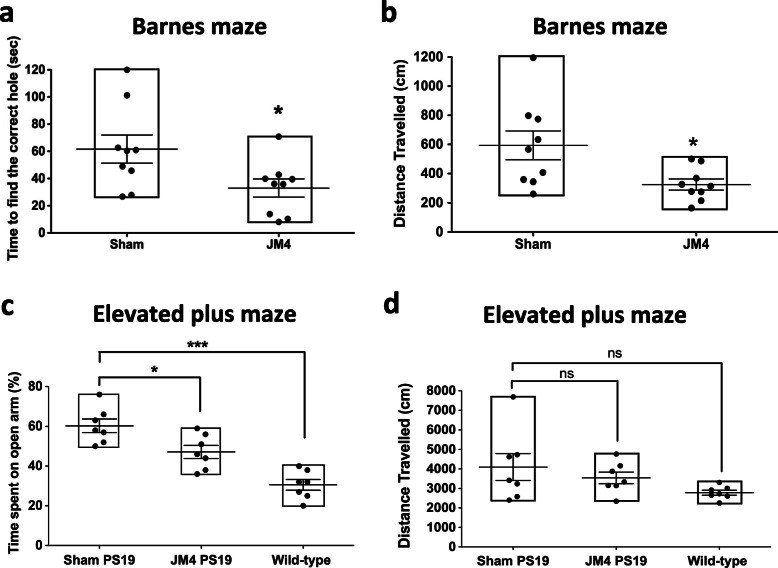
Chronic JM4 treatment rescued behavioral abnormalities in PS19 mice. **a** JM4-treated PS19 mice at 7 months of age took less time to find the target hole than PBS sham-treated group in the Barnes maze test (*p < 0.05, two-tailed unpaired *t* test). **b** PBS sham-treated PS19 mice traveled a longer distance to find the target hole in the Barnes maze test (*p < 0.05, two-tailed unpaired *t* test). **c** PS19 mice spent more time on the open arm than the wild-type mice suggesting PS19 mice have less anxiety-like behavior when compared to wild-type mice (***p < 0.001, PBS sham-treated PS 19 mice vs. wild-type mice, one-way ANOVA followed by Tukey’s multiple comparison test). Chronic JM4 treatment significantly reduced this behavioral abnormality in PS19 mice (*p < 0.05, PBS sham-treated PS19 mice vs. JM4**-**treated PS19 mice). **d** There was no significant difference in total distance traveled among PBS sham-treated PS19 mice, JM4-treated PS19 mice, and wild-type control (ns, not significant; one-way ANOVA followed by Tukey’s multiple comparison test)

For assessing spatial memory, we used the Barnes maze test, which relies on the innate preference of mice for dark, enclosed spaces [39]. At 7 months of age, JM4-treated PS19 mice took less time than PBS sham-treated PS19 mice to find a target hole (JM4-treated group 33.0 ± 6.6 s, PBS sham-treated group 61.6 ± 10.7 s, *n* = 9 for each group) suggesting that JM4 treatment can rescue memory impairment in PS19 mice (Fig. 2a). The PBS sham-treated PS19 mice also traveled a longer distance to find a target hole (JM4-treated group 325.5 ± 37.5 cm, PBS sham-treated group 593.2 ± 98.7 cm, *n* = 9 for each group), suggesting that PBS sham-treated PS19 mice took more time to find a target hole due to memory impairment rather than due to motor deficit (Fig. 2b).

Next, we carried out elevated plus maze test to assess anxiety level since previous studies found PS19 mice had reduced anxiety-like behavior [35]. We found that PS19 mice spent more time in the open arm than wild-type mice at 8 months of age. This result is consistent with reduced anxiety-like behavior in PS19 mice compared to wild-type mice. Importantly, chronic JM4 treatment significantly reduced this behavioral deficit in PS19 mice (percentage of time spent in the open arms: JM4-treated group 47 ± 3.3%, PBS sham-treated group 60 ± 3.4%, wild-type control 31 ± 2.7%, *n* = 7 for each group) (Fig. 2c). Again, motor deficit of PS19 mice at this age did not interfere with test performance, as there were no significant differences in the distance traveled among different groups (JM4-treated group 3543 ± 300 cm, PBS sham-treated group 4099 ± 689 cm, wild-type control 2784 ± 126 cm, *n* = 7 for each group) (Fig. 2d). Instead, there is a trend that the total distance traveled by PBS sham-treated mice is greater than that by wild-type mice, which is consistent with previous findings that PS19 mice are hyperactive [35].

### Chronic JM4 treatment blocks neuronal loss in PS19 mice

**Fig. 3:**
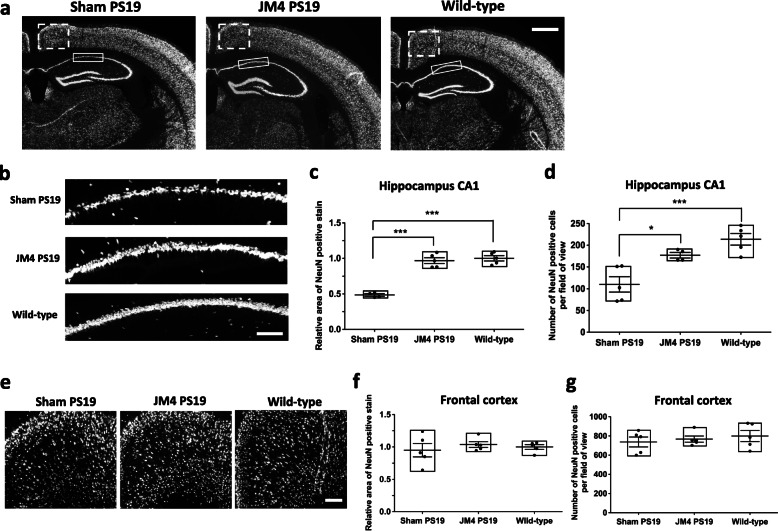
Chronic JM4 treatment blocked neuron loss in PS19 mouse brains. **a** Images of coronal sections of mouse brains containing the hippocampus at 10 months of age showing NeuN immunoreactivity. × 2 objective. Rectangles mark CA1 region and dashed rectangles mark frontal cortex region selected for quantification. Scale bar 500 μm. **b** Magnified images of the hippocampus CA1 region showing NeuN immunoreactivity. × 10 objective. Scale bar 100 μm. There is marked decrease in the number of neuronal cell bodies stained by NeuN antibody in PBS sham-treated PS19 mice brain compared to JM4**-**treated PS19 mice brain. **c** Quantification of NeuN immunoreactivity in the hippocampus CA1 region. The values were normalized to the area in wild-type control mice brain sections (***p < 0.001, PBS sham-treated PS19 mice vs. wild-type mice, and PBS sham-treated PS19 mice vs. JM4**-**treated PS19 mice, one-way ANOVA followed by Tukey’s multiple comparison test). **d** Quantification of the number of NeuN positive neurons per a field of view in the hippocampus CA1 region (***p < 0.001, PBS sham-treated PS19 mice vs. wild-type mice, and *p < 0.05 PBS sham-treated PS19 mice vs. JM4**-**treated PS19 mice, one-way ANOVA followed by Tukey’s multiple comparison test). **e** Magnified images of the frontal cortex region showing NeuN immunoreactivity. × 10 objective. Scale bar 100 μm. **f** Quantification of NeuN immunoreactivity in the frontal cortex. The values were normalized to the area in wild-type control mice brain sections. No statistically significant differences were noted. **g** Quantification of the number of NeuN positive neurons per a field of view in the frontal cortex. No statistically significant differences were noted

**Fig. 4:**
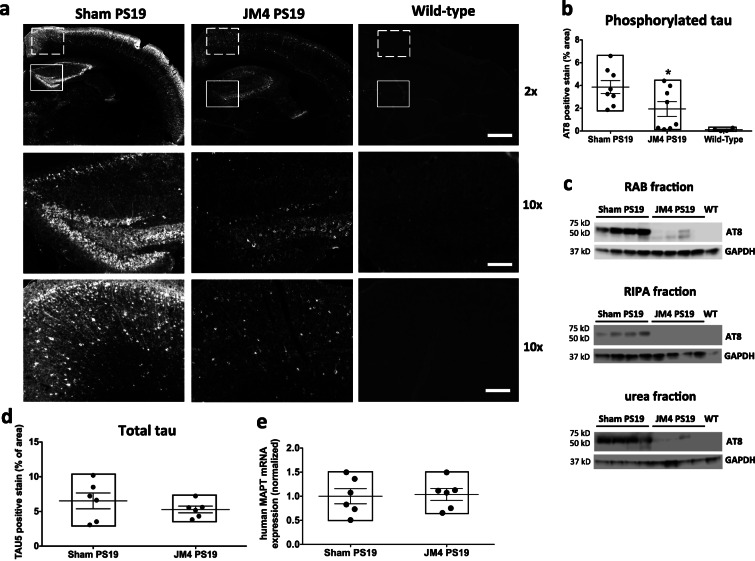
Chronic JM4 treatment reduced hyperphosphorylated tau aggregates in PS19 mouse brains. **a** Top row: Images of coronal sections of mouse brains containing the hippocampus at 10 months of age showing AT8 immunoreactivity. Rectangles mark dentate gyrus region and dashed rectangles mark frontal cortex region selected for quantification. × 2 objective. Scale bar 500 μm. Middle row: Magnified images of the hippocampus dentate gyrus region showing AT8 immunoreactivity. × 10 objective. Scale bar 100 μm. Bottom row: Magnified images of the frontal cortex region showing AT8 immunoreactivity. × 10 objective. Scale bar 100 μm. The left panels show abundant strongly positive hyperphosphorylated tau aggregates recognized by the AT8 antibody in PBS sham-treated PS19 mouse brain. The middle panels show dramatically reduced levels of AT8 immunoreactivity in JM4**-**treated PS19 mouse brain. The right panels show essentially no AT8 immunoreactivity in wild-type mouse brain. **b** Quantification of AT8 immunoreactivity in the hippocampus dentate gyrus region (*p < 0.05, one-way ANOVA followed by Bonferroni test to compare JM4-treated group and PBS sham-treated group). **c** Western blots of mouse brain homogenates of PBS sham-treated PS19 mice, JM4**-**treated PS19 mice, and wild-type (WT) mice showing the immunoreactivity of RAB fraction, RIPA fraction, and urea fraction detected by AT8 antibody. GAPDH served as a loading control. **d** Quantification of TAU-5 immunoreactivity in the brain slices containing the hippocampus. No statistically significant difference was noted on two-tailed unpaired *t* test. **e** Quantitative RT-PCR analysis of human MAPT mRNA levels relative to mouse GAPDH mRNA level in 10 months of age mouse brain lysates. The data was normalized to the mean value of the PBS sham-treated group. No statistically significant difference was noted on two-tailed unpaired *t* test

**Fig. 5:**
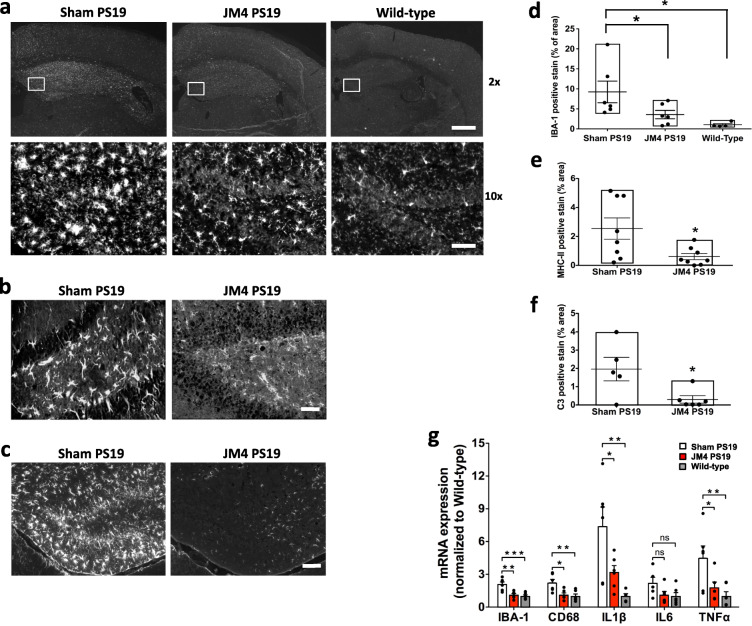
Chronic JM4 treatment reduced microglial activation in PS19 mouse brains. **a** Top row: Images of coronal sections of mouse brains containing the hippocampus at 10 months of age showing IBA-1 immunoreactivity. Rectangles mark dentate gyrus region. × 2 objective. Scale bar 500 μm. Bottom row: Magnified images of the hippocampus dentate gyrus region showing IBA-1 immunoreactivity. × 10 objective. Scale bar: 50 μm. **b** Magnified images of mouse brain sections containing the hippocampus dentate gyrus region at 10 months of age showing strong MHC II immunoreactivity displaying numerous reactive microglia with thickened appendages in PBS sham-treated P19 mouse brain (left). In contrast, there is diminished MHC II signal in the JM4**-**treated PS19 mouse brain (right). Scale bar 50 μm. **c** Magnified mages of mouse brains sections containing the hippocampus dentate gyrus region at 10 months of age showing strong C3 immunoreactivity in PBS sham-treated P19 mouse brain (left). In contrast, there is diminished C3 immunoreactivity in the JM4**-**treated PS19 mouse brain (right). Scale bar 100 μm. **d** Quantification of IBA-1 immunoreactivity in the hippocampus dentate gyrus region (*p < 0.05, one-way ANOVA followed by Bonferroni test to compare JM4-treated group and PBS sham-treated group and to compare wild-type and PBS sham-treated group). **e** Quantification of MHC II immunoreactivity (*p < 0.05, two-tailed unpaired *t* test). **f** Quantification of C3 immunoreactivity (*p < 0.05, two-tailed unpaired *t* test). **g** Quantitative RT-PCR analysis of mouse IBA-1, CD68, IL1β, IL6, and TNFα mRNA levels relative to mouse GAPDH mRNA level in 10 months of age mouse brain lysates (*p < 0.05, **p < 0.01, ***p < 0.001, ns, not significant; one-way ANOVA followed by Bonferroni test to compare JM4-treated group and PBS sham-treated group or to compare wild-type and PBS sham-treated group). The data was normalized to the mean value of wild-type

A subset of PS19 mice that were treated with JM4 from 1 month old and underwent behavioral testing was sacrificed at 10 months old and used for immunohistochemical analysis (Figs. 3, 4, 5).

PS19 mice develop neuronal loss and brain atrophy by 8 months of age, mainly in the hippocampus, and then neuronal loss becomes apparent in other brain regions including neocortex by 12 months of age [18]. Thus, the brain ﻿sections of 10-month-old PS19 mice were examined for the effects of JM4 treatment on neuronal loss by quantifying neuronal nuclei (NeuN) staining in hippocampal and cortical sections. JM4 treatment led to a significantly greater area of NeuN immunoreactivity in the hippocampus CA1 region compared to PBS sham-treatment in PS19 mice (relative NeuN immunoreactive area: PBS sham-treated PS19 mice 0.49 ± 0.02, JM4-treated PS19 mice 0.97 ± 0.04, wild-type mice 1.00 ± 0.04. *n* = 5 for each group) (Fig. 3a–c). To exclude the possibility that the change in the area of NeuN immunoreactivity was due to a change in size or in morphology of neurons, we also counted the number of NeuN positive neurons and found similar results (the number of NeuN positive neurons per a field of view: PBS sham-treated PS19 mice 110.2 ± 17.8, JM4-treated PS19 mice 176.9 ± 6.4, wild-type mice 213.6 ± 13.2, *n* = 5 for each group) (Fig. 3d). These results suggest that chronic JM4 treatment prevented neuronal loss in the hippocampus of PS19 mice. There was no significant difference in the area of NeuN immunoreactivity or the number of NeuN positive neurons in the frontal cortical sections of 10 months old PS19 mice vs. 10 months old wild-type mice (relative NeuN immunoreactive area: PBS sham-treated PS19 mice 0.95 ± 0.10, JM4-treated PS19 mice 1.04 ± 0.04, wild-type mice 1.00 ± 0.04; the number of NeuN positive neurons per a field of view: PBS sham-treated PS19 mice 737.4 ± 51.9, JM4-treated PS19 mice 767.5 ± 32.1, wild-type mice 799.5 ± 57.4, *n* = 5 for each group) (Fig. 3e–g). This finding is consistent with a published report that neuronal loss occurs at later time in the neocortex compared to the hippocampus in PS19 mice [18].

### Chronic JM4 treatment reduced phosphorylated tau aggregates in PS19 mice

Accumulation of hyperphosphorylated tau aggregates, in addition to neuronal loss, is a pathologic hallmark of PS19 mice [18]. Thus, the brain sections of 10-month-old mice were also evaluated for tau aggregates by immunohistochemistry. We used AT8 tau antibody, which recognizes tau protein phosphorylated at both serine 202 and threonine 205, but lacks cross-reactivity with unphosphorylated tau [40]. JM4 treatment significantly reduced AT8 immunoreactivity per unit area in the hippocampus compared to PBS sham-treatment in PS19 mice, while brains of wild-type mice showed essentially no AT8 immunoreactivity (percentage of AT8 immunoreactive area: PBS sham-treated PS19 mice 3.86 ± 0.58, *n* = 8, JM4-treated PS19 mice 1.93 ± 0.64, *n* = 8, wild-type mice 0.11 ± 0.06, *n* = 4) (Fig. 4a, b). Qualitatively similar results are seen in the cortex, but we focused the analysis of tau immunoreactivity and subsequent analysis of neuroinflammation on the hippocampus because the hippocampus was the main brain region with neuronal loss in 10-month-old PS19 mice. We also carried out Western blot analysis of total brain homogenates of these mice. Similar to immunohistochemistry of brain sections, we found that chronic JM4 treatment decreased phosphorylated tau level in soluble RAB fraction, RIPA fraction, and insoluble urea fraction of mouse brain homogenates (Fig. 4c).

To exclude the possibility that JM4 treatment reduced AT8 immunoreactivity by suppression of human tau transgene expression in PS19 mice, we also immunostained the hippocampal sections with TAU-5 antibody, which stains total tau—both unphosphorylated and phosphorylated tau species [41]. Unlike the finding with AT8 tau antibody, there was no significant difference in TAU-5 immunoreactivity between the hippocampal sections of PS19 mice treated with JM4 and sham-treated with PBS (percentage of TAU-5 immunoreactive area: PBS sham-treated PS19 mice 6.53 ± 1.14, JM4**-**treated PS19 mice 5.29 ± 0.48. *n* = 6 for each group) (Fig. 4d).

We also carried out quantitative RT-PCR of the PS19 mouse brain lysate to assess further whether chronic JM4 treatment altered human tau transgene expression level. There was no significant difference in human MAPT mRNA expression between PS19 mice treated with JM4 and sham-treated with PBS (normalized ratio of human MAPT/mouse GAPDH expression: PBS sham-treated PS19 mice 1.00 ± 0.16, JM4**-**treated PS19 mice 1.04 ± 0.12**,** *n* = 6 for each group) (Fig. 4e). Taken together, these results show that JM4 treatment leads to a reduction of hyperphosphorylated tau aggregates without affecting expression of tau transgene in PS19 mice.

### Chronic JM4 treatment reduced microglial activation in PS19 mice

Since we hypothesized that the beneficial effect of JM4 was, at least in part, due to its anti-inflammatory/immunomodulatory properties leading to downregulation of microglial activation, we first assessed effects of chronic JM4 treatment on a microglial marker, IBA-1, in brain sections of these mice using immunohistochemistry. We found that IBA-1 immunoreactivity per unit area in the hippocampus was elevated in PS19 mice compared to wild-type control; more importantly, JM4 treatment led to a reduction in IBA-1 immunoreactivity compared to PBS sham-treated group in PS19 mice (percentage of IBA-1 immunoreactive area: PBS sham**-**treated PS19 mice 9.24 ± 2.71, *n* = 6, JM4**-**treated PS19 mice 3.59 ± 1.05, *n* = 6, wild-type mice 1.04 ± 0.31, *n* = 4) (Fig. 5a, d).

Because IBA-1 is thought to be a pan microglia marker identifying all microglia [42], we next assessed a more specific marker of microglial activation, the major histocompatibility complex II (MHC II) proteins in the hippocampus. Microglia normally express low levels of MHC II; however, in inflammatory or neurodegenerative conditions, activated microglia highly upregulate MHC II [43]. Chronic JM4 treatment led to a lower level of MHC II immunoreactivity per unit area in the PS19 mouse hippocampus compared to PBS sham-treated PS 19 mouse hippocampus, suggesting JM4 treatment led to reduced microglial activation (percentage of MHC II immunoreactive area: PBS sham-treated PS19 mice 2.54 ± 0.74, JM4**-**treated PS19 mice 0.61 ± 0.22. *n* = 8 for each group) (Fig. 5b, e).

Recently, the complement cascade, which is a major effector pathway of microglial activation, has been shown to play an important role in tau pathology in AD brain as well as the mouse model of tauopathy [7–10]. The complement cascade converges on a central component C3, whose fragments interact with their receptors, C3aR and CR3, to enact downstream immune effects such as phagocytosis [44]. For this reason, we carried out immunohistochemistry of these brain sections with an antibody against C3. Similar to the results with MHC II, we found that chronic JM4 treatment led to lower levels of C3 immunoreactivity in the PS19 mouse hippocampus compared to PBS sham-treated PS 19 mouse hippocampus (percentage of C3 immunoreactive area: PBS sham-treated PS19 mice, *n* = 5: 1.96 ± 0.65, JM4**-**treated PS19 mice, *n* = 6: 0.30 ± 0.20) (Fig. 5c, f).

We also carried out quantitative RT-PCR of the PS19 mouse brain lysate to assess further whether chronic JM4 treatment reduced expression levels of microglial markers. Consistent with immunohistochemistry data, we found that JM4 treatment led to a reduction in IBA-1 mRNA expression compared to PBS sham-treated group in PS19 mice (normalized IBA-1 mRNA expression level: PBS sham**-**treated PS19 mice 2.08 ± 0.22, JM4**-**treated PS19 mice 1.10 ± 0.13, wild-type mice 1.00 ± 0.12, *n* = 5–6) (Fig. 5g). We also assessed mRNA level of CD68 (cluster of differentiation 68), a phagocytic microglia marker that has been found to be elevated in PS19 mouse brains [8, 9]. We found that CD68 mRNA expression was elevated in PS19 mouse brains compared to wild-type mouse brain, and JM4 treatment led to a reduction in CD68 mRNA expression compared to PBS sham-treated group in PS19 mice (normalized CD68 mRNA expression level: PBS sham**-**treated PS19 mice 2.22 ± 0.32, JM4**-**treated PS19 mice 1.12 ± 0.18, wild-type mice 1.00 ± 0.20, *n* = 5–6) (Fig. 5g).

It has been shown that levels of proinflammatory cytokines including IL1β, IL6, and TNFα were elevated in PS19 mouse brains [9, 45]. Therefore, we additionally assess the expression levels of IL1β, IL6, and TNFα mRNAs using quantitative RT-PCR of the PS19 mouse brain lysate. We also found that IL1β and TNFα mRNAs levels were elevated in PS19 mouse brains and they were reduced by chronic JM4 treatment. There was a trend toward increase of IL6 mRNA level in PS19 mouse and reduction by chronic JM4 treatment, but it did not reach the statistical significance (normalized IL1β mRNA expression level: PBS sham**-**treated PS19 mice 7.38 ± 1.79, JM4**-**treated PS19 mice 3.19 ± 0.61, wild-type mice 1.00 ± 0.23, *n* = 5–6; normalized IL6 mRNA expression level: PBS sham**-**treated PS19 mice 2.18 ± 0.55, JM4**-**treated PS19 mice 1.11 ± 0.32, wild-type mice 1.00 ± 0.33, *n* = 5–6; normalized TNFα mRNA expression level: PBS sham**-**treated PS19 mice 4.48 ± 1.12, JM4**-**treated PS19 mice 1.79 ± 0.50, wild-type mice 1.00 ± 0.41, *n* = 5–6) (Fig. 5g). Taken together, these results show that chronic JM4 treatment leads to a reduction of activated microglia and downstream proinflammatory cytokines and complement components in PS19 mouse brains.

## Discussion

In our study, we found that chronic treatment with JM4, a small peptide derived from the first loop of human EPO, delayed the onset of neurological deficit and prolonged lifespan in the PS19 mouse, an animal model of tauopathy. However, it is clear from our data that JM4 treatment does not prevent the disease completely, i.e., JM4 treatment is not a cure for tauopathy. With a current incomplete understanding of all the contributing factors to the pathogenesis of tauopathies including AD and related dementias, we suspect that finding a drug candidate that can completely prevent the disease process or cure the disease will be extremely difficult. However, even a candidate that can delay significantly the progression of the disease would be enormously beneficial to reduce economic and human cost of treating tauopathies.

In addition to clinical benefit, we showed that chronic JM4 treatment reduced both neuronal loss (neurodegeneration) and microglial activation manifested as IBA-1 and MHC II induction as well as complement component C3 elevation (neuroinflammation) in the brains of 10 months old PS19 mice. We also see decreases in selected proinflammatory cytokine expressions by chronic JM4 treatment. Current evidence is overwhelmingly clear that neuroinflammation mediated by microglial activation is critical in pathogenesis of tauopathies including AD and related dementias [1–3]. The primary neuroinflammation received the bulk of attention recently, but the secondary neuroinflammation in response to neuronal loss in neurodegenerative condition is also likely occurring [4]. Our current results cannot distinguish whether JM4 acts directly on neuroinflammation or whether the decrease in inflammation is a secondary effect of reducing neurodegeneration because both neuroinflammation and neurodegeneration are observed at 10 months of age in these mice. We raised a possibility that JM4 treatment could also provide direct neuroprotection in this model of tauopathy because we previously showed that JM4 prevented apoptotic neuronal loss in acute traumatic brain injury in mice [33]. In future studies, we will carry out neuropathological examination at 6 months of age, when PS19 mice exhibit cognitive changes and microglial activation, but no overt neurodegeneration [18]. Assessing at this time point will allow us to establish whether JM4 affects neuroinflammation directly, as opposed to reducing neurodegeneration, which then produces a secondary effect on neuroinflammation.

Regardless whether JM4 has direct effect on neuroinflammation, secondary effect on neuroinflammation, or both, merely stating that JM4 exerts beneficial effects by preventing microglial activation is an over-simplified explanation. It has become apparent that microglia are not a monolithic group of immune cells that cause destruction in the CNS under neurodegenerative conditions, but subsets of microglia also have protective roles [5, 6]. They have heterogeneous molecular signatures, and thus, the role of microglia in neurodegenerative diseases including AD is diverse depending on microglial gene expression patterns [46]. In addition, the role of microglia is profoundly dependent on the stage of disease progression [47]. It appears that in early stages of disease, microglia attempt to clear toxic stimuli, but at later stages, persistent toxic stimuli lead to exaggerated proinflammatory responses by microglia [48]. Therefore, it is more likely the beneficial effects of JM4 are due to restoring normal pattern and balance of microglial population and immune homeostasis rather than simply preventing microglial activation in the CNS. In future studies, JM4 modulation of microglial properties in mouse models of tauopathies including AD needs to be investigated more thoroughly in different stages of the disease process and in different subsets of microglia delineated by various microglial markers or gene expression profiling.

Although JM4 crosses the blood brain barrier and affects microglial activation in the CNS, it is possible that JM4’s beneficial effect is not exclusively due to its effect on the immune system within the CNS. Emerging data suggest that an interaction between the central and peripheral immune system is also important in neurodegenerative diseases. For example, there are increased numbers of CD4+ IL17+ T helper cells (CD4 Th-17) in peripheral blood of patients with Parkinson’s disease and IL17 blockage rescues cell death [49]. In therapeutics for multiple sclerosis, a demyelinating disease of the CNS, medications such as fingolimod exert their beneficial effects mainly by modulating the peripheral immune system rather than directly affecting the immune system in the CNS [50]. Thus, the effect of JM4 on the immune system within the CNS and its potential effect on the peripheral immune system are not mutually exclusive. Indeed, in EAE mice, JM4 normalizes peripheral immune activation by reducing the total number of mononuclear cells and CD11c + dendritic cells in the lymph nodes, modulating the adaptive immune system by decreasing numbers of CD4 Th-17, and expanding CD4+ Foxp3+ regulatory T cells (Treg) in the spleen [33]. Therefore, it will be important to assess the effects of JM4 on the peripheral immune system in addition to the immune system within the CNS of PS19 mice to define more completely the mechanism of action of JM4.

Our results show that chronic JM4 treatment decreased the hyperphosphorylated tau aggregates recognized by the AT8 antibody without affecting the expression of tau transgene in PS19 mice. How does JM4 reduce tau aggregates? We favor an indirect effect of JM4 in reducing tau aggregates via its modulation of microglia, as there is strong evidence for a critical role of microglia in triggering tau aggregates in tauopathies including AD. For example, genetic deletion of a complement component, C3aR, leads to the rescue of tau pathology in PS19 mice and signal transducer and activator of transcription 3 (STAT3) is a downstream target of C3-C3aR signaling [9]. Moreover, activated microglia may induce tau pathology via promoting hyperphosphorylation of tau through their production of IL1 and activation of neuronal p38-MAPK pathway and this process is regulated by the microglial-specific fractalkine receptor (CX3CR1) [51, 52]. Assessing whether chronic JM4 treatment could inhibit neuronal p38-MAPK pathway and enhance CX3CR1 as well as modulating the STAT3 pathway in the microglia should provide us mechanisms by which chronic JM4 treatment reduces tau pathology. The reduction of tau aggregates by JM4 can be achieved by additional mechanisms, which include inhibiting assembly of tau, disrupting tau aggregates, enhancing cellular mechanisms to eliminate toxic forms of tau, stabilizing microtubules, and inhibiting the spread of toxic forms of tau [53].

 A crucial role of neuroinflammation in pathogenesis of tauopathies has been known for some times, and there has been a number of drug candidates that target neuroinflammation—both novel drug candidates and repurposing of existing drugs—under investigation [13]. For example, multiple clinical trials investigated the efficacy of existing anti-inflammatory drugs, aspirin or non-steroidal anti-inflammatory drugs (NSAIDs) in AD, but unfortunately, no trials have been successful thus far [54]. On the other hand, novel drug candidates usually require a long preclinical development time line. JM4 is more advantageous than other novel drug candidates, as it already went through the entire preclinical development process including GMP quality JM4 production and short-term toxicology studies supported by the NIH. This decision was based on our previous findings in EAE mice treated with JM4 as preclinical studies for use of JM4 in multiple sclerosis therapy. The FDA is currently evaluating our investigational new drug (IND) application for human use. Given this advanced preclinical drug development timeline, JM4 should be further investigated as a new therapeutic agent for tauopathies including AD and related dementias.

## Limitations

Limitations of the present study include that we only tested a single dose of JM4 (10 μg) in the PS19 mouse. That particular dose was selected based on our previous results with EAE and acute traumatic brain injury model in mice [33, 34], but it is possible that an optimal effective dose of JM4 for the PS19 mouse may be different. A dose response study of JM4 on the PS19 mouse is needed. Furthermore, we carried out our present study on female PS19 mice only to avoid potential sex differences in response to treatment. However, it was recently reported that male PS19 mice exhibited greater behavioral deficit and neuropathological changes [55]. Therefore, male PS19 mice may be served as a more sensitive platform to assess therapeutic potential of JM4 in tauopathies including AD.

## Conclusion

In this preclinical in vivo study, we assess the effects of JM4, a 19’mer peptide derived from the first loop of human EPO, on the PS19 mouse, an animal model of tauopathy. We show that daily subcutaneous treatment with JM4 initiated before disease onset reduced neurologic deficits, prolonged lifespan, rescued memory impairment, and decreased neuronal loss, NFT accumulation, and microglial activation in PS19 mice. This is one of the first studies showing beneficial effects of EPO or its derivatives in a mouse model of tauopathy. These results demonstrate that JM4 is a potential novel therapeutic agent for the treatment of tauopathies including AD and related dementias.

## Data Availability

All data sets used and analyzed during the current study are available from the corresponding author on reasonable request.

## Abbreviations

AD: Alzheimer’s disease
CD68: Cluster of differentiation 68
CNS: Central nervous system
EAE: Experimental autoimmune encephalomyelitis
EPO: Erythropoietin
FTD: Frontotemporal dementia
GAPDH: Glyceraldehyde 3-phosphate dehydrogenase
HPLC: High performance liquid chromatography
IACUC: Institutional Animal Care and Use Committee
IBA-1: Ionized calcium-binding adapter molecule 1
IL1β: Interleukin 1 beta
IL6: Interleukin 6
IND: Investigational new drug
MHC II: Major histocompatibility complex II
NFT: Neurofibrillary tangles
NeuN: Neuronal nuclei
PBS: Phosphate-buffered saline
RAB: Reassembly
RIPA: Radioimmunoprecipitation assay
RT-PCR: Reverse-transcription polymerase chain reaction
STAT 3: Signal transducer and activator of transcription 3
TNFα: Tumor necrosis factor alpha
